# The Impact of Clinical Pharmacy Services in a Tertiary Care Center Specialized in Pediatric Hemato-Oncology

**DOI:** 10.3390/children9040479

**Published:** 2022-03-31

**Authors:** Christina Gradwohl, Gernot Engstler, Martina Anditsch, Herbert Pichler, Gunar Stemer

**Affiliations:** 1 St. Anna Children’s Hospital, Department of Paediatrics, Medical University of Vienna, 1090 Vienna, Austria; gernot.engstler@stanna.at (G.E.); herbert.pichler@stanna.at (H.P.); 2 Pharmacy Department, University Hospital AKH, 1090 Vienna, Austria; martina.anditsch@akhwien.at (M.A.); gunar.stemer@akhwien.at (G.S.)

**Keywords:** pediatrics, hematopoietic stem cell transplantation, hematology, oncology, clinical pharmacy services, drug-related problems

## Abstract

Clinical pharmacy services (CPS) have shown beneficial effects on several outcome measures in hospital patients, including the reduction of drug-related problems (DRP) and of therapy costs. Less is known about the impact of CPS in pediatric haemato-oncology, even though this patient population is highly susceptible to DRP. CPS were implemented in a tertiary care children’s hospital specialized in hemato-oncology and hematopoietic stem cell transplantation. The main outcome measures were type and number of DRP, type and number of pharmaceutical interventions (PI), their acceptance rate, and their clinical significance and economic benefit. During 6 months and 32 ward rounds, 275 DRP were identified and addressed by PI. The acceptance of PI was high (73.4%), and up to 80% of PI were rated as very significant or significant by independent external raters. The estimated therapy cost reductions were substantial, approaching at least EUR 54,600 for avoided follow-up costs. Conclusion: CPS improve medication safety in pediatric hemato-oncology and may reduce therapy costs.

## 1. Introduction

Compared to adults, children appear to have a higher risk for drug-related problems (DRP) and are subject to higher rates of medication errors [1]. A high proportion of drugs being administered off-label or off-license complicates optimal pediatric pharmacotherapy and jeopardizes medication safety in children, as systematically generated data on correct dosage, side effects, or contraindications are lacking [2].

Children treated for hemato-oncological diseases or those receiving a hematopoietic stem cell transplantation (HSCT) are specifically susceptible to DRP, in view of the fact that intense chemotherapy protocols frequently result in the need of a broad range of supportive care, including the administration of drugs with a narrow therapeutic index [2,3]. In addition, pre-existing comorbidities or treatment-related toxicities regularly require dose adjustments and close monitoring of drug effects.

Pediatric clinical pharmacy services (CPS) are increasingly established and introduced to daily patient care in tertiary care institutions to assist clinicians in maintaining the appropriate use of drugs [4]. Recent publications outline several positive outcome-measures (e.g., reduced number of DRP, decreased length of hospital stay and cost reductions) for pediatric CPS [4]. Hence, clinical pharmacists should be considered as an integral part of multidisciplinary patient care teams in contemporary pediatric hospitals.

The aim of this work is to present the benefits of a newly established CPS in a tertiary care children’s hospital specialized in pediatric hemato-oncology and HSCT in Vienna, Austria.

## 2. Materials and Methods

A 6-month CPS implementation and evaluation study on two wards (A: hemato-oncology, 11 beds; B: HSCT unit, 10 beds) was conducted between June and December 2020 at the St. Anna Children’s Hospital in Vienna, Austria. Both wards are intermediate-care units, which include patients who are taking part in (inter-)national treatment studies and phase I, II, and III studies. The responsible clinical pharmacist (CP) had 4 years of professional experience and received pre-training by the CPS team of the University Hospital AKH, Vienna.

CPS were provided as twice-weekly ward round participation, with preceding in-depth medication reviews of all admitted patients. DRP and pharmaceutical interventions (PI) were quantified, and their acceptance rate determined. Documentation was performed along established criteria [5,6]. There were no direct CP–patient/parents interactions, nor any changes in pharmacotherapy initiated by the CP on her own. PI were self-assessed for clinical significance using the Hatoum scale [7]. An external blinded validation of self-assessment was performed for a 10% random sample of PI by 4 independent expert raters (2 senior hemato-oncologists, 2 clinical pharmacists). Inter-rater reliability was calculated using Spearman’s correlation coefficient [8].

Economic benefit of CPS was retrospectively approximated, first by rating accepted PI associated with drug therapy cost reductions and adding up costs if drug therapy had continued for another 5 days without PI (based on health insurance price) and second by valuing follow-up costs that were considered as avoidable through the prevention of side effects [6]. Avoided follow-up costs were calculated based on the method of Zuba et al. (2016), which was developed for a comparable CPS evaluation project in Austrian hospitals and financed by public-law funds of the Austrian Ministry of Health. First, the number of identified particularly problematic DRP was calculated. Second, the number of avoided side effects as a result of managing these DRP by accepted PI was calculated. Third, the number of avoided side effects was multiplied by the approximated costs of a side effect. The costs of CPS provision only included labor costs. Calculations were performed using Microsoft Excel and SPSS. Open feedback interviews were voluntarily conducted with senior physicians and head nurses to reflect upon the quality of CPS and to decide upon an expansion of the services.

Main outcome measures were type and number of DRP, type of PI, and their acceptance rate. Further outcome parameters were clinical significance of PI and economic benefit.

## 3. Results

During 32 ward rounds and after 172 preceding medication reviews, the CP performed PI addressing 275 DRP in 40 pediatric patients (median age 8.0 years (0.4–24), 35.1% girls). The mean number of DRP per ward round was 8.6 ± 3.5, and the mean number of DRP per medication review during a ward round was 1.8 ± 1.2. The acceptance rate, recorded for 67% of PI (excluding informational and organizational contributions), was 73.4%. The most frequent DRP regarded drug monitoring (16.7%), need for information and therapy discussion (16.4%), or drug interactions (16%). The most frequent PI involved drug monitoring (24.4%), drug information (22.2%), and dose adjustments (20%). The Top 10 drugs involved in PI are shown in Table 1. 

The CP assessed 64% of PI as clinically ‘significant’ or ‘very significant’. ‘Significant‘ interventions bring care to a more acceptable and appropriate level, whereas ‘very significant‘ interventions are qualified by potential or existing major organ dysfunction [7]. In addition, 20% of the interventions were classified as ‘not significant‘ due to informational contributions not specifically related to the patient in question, and 16% as ‘somewhat significant‘ (benefit could be neutral, depending on professional interpretation). No interventions were classified as ‘adversely significant‘. The correlation between external and self-assessment was statistically significant (Spearman Rho 0.637, *p* < 0.0001). Hemato-oncologists assessed 80% of PI as ‘significant’ or ‘very significant’.

The costs of CPS were EUR 7200. The CPS impact on drug therapy costs was estimated for three-fourth of PI (excluding informational and organizational contributions), whereof 41.1% were classified as cost-reducing. This led to estimated cost reductions of EUR 5660.

Follow-up costs were calculated based on the method of Zuba et al. (2016) [6]. According to Vermeulen et al. (2014) [9], 28% of particularly problematic DRP (e.g., overdosing, contraindicated combination) lead to side effects. In this study, accepted PI addressed 49 of such particularly problematic DRP, which corresponds to the calculated avoidance of 14 side effects. The costs of the side effects were evaluated between EUR 1300 and 2500 [6]. Hence, we estimate that the PI might have avoided follow-up costs between EUR 18,200 and 35,000. Furthermore, the CP detected 28 side effects. Assuming that the PI led to a better side effect management, which reduced the costs by EUR 1300 per side effect [6], further follow-up cost reductions of EUR 36,400 were estimated.

The narrative feedback of three physicians and one nurse revealed that the CPS were appreciated and that the expansion of CPS is desired.

## 4. Discussion

CPs as part of the multidisciplinary team in a pediatric hemato-oncology ward or a HSCT unit are able to detect DRP and suggest clinically significant PI with a high rate of acceptance by the prescribing physicians. Consequently, DRP such as preventable side effects will be avoided [9]. For example, a study tracking pre- and post-pharmacist involvement shows that CPS result in a reduction of serious medication errors in critically ill hospitalized pediatric patients [10]. The implementation of CPS also leads to the prevention of further DRP [11]. We hypothesize that this is due to a knowledge transfer by the ward team that results in a learning curve. The overall reduction in DRP is capable to beneficially affect patient outcomes as it is considered a surrogate for reduced hospital admissions [12].

During a study period of 6 months, the responsible CP initiated an average of nearly nine PI per ward round. This is comparable to the results of Prot-Labarthe et al., who reported 16.9 interventions/day (mean) initiated in two wards [13]. The acceptance rate of nearly 75% is high but slightly lower as compared to other studies that reported rates of 86–93% [13,14]. Conceivable explanations would be a learning curve of the CP. Self-assessment showed 64% of PI deemed as significant or higher, whereas hemato-oncologists tended to rate the significance of PI higher (80%). These findings demonstrate that self-assessment did not overestimate the significance of PI for clinical practice.

The study indicates that CPS are able to reduce the estimated drug therapy and follow-up costs. The actual value of avoided follow-up costs could be higher, as other DRP than suggested by Vermeulen et al. were not included in the calculation. Further follow-up costs such as hospital readmissions or an increased quality of life were not investigated. Overall, the estimated economic benefit of CPS was at least eight-fold higher than its costs.

The conducted study also comes with limitations. The patient population was highly specific, and the patient number was small. No comparisons of clinical outcomes such as frequency of medication errors resulting in harm or time to readmission were made between the pre- and the post-intervention phase. The prevention of DRP because of a successful implementation of CPS is only a surrogate for improved patient outcomes. The scope of the CPS was narrow, as additional patient care activities (e.g., patient education) were lacking. The cost reductions were estimated only retrospectively, based on extrapolations of published data. No other follow-up cost reductions than those caused by the avoidance of side effects were calculated.

## 5. Conclusions

CPS in pediatric haemato-oncology wards or HSCT units may substantially improve drug therapy and increase medication safety by lowering the frequency of DRP. From a management perspective, the economic benefit of CPS was recognizable and resulted in an expansion of services in the investigated hospital.

## Figures and Tables

**Table 1:** Top 10 drugs involved in detected drug-related problems (DRP) and corresponding examples of DRP and pharmaceutical interventions (PI).

Drug	*n*	Example of DRP	DRP	Example of PI	PI
Pantoprazole	25	Reduced absorption of Posaconazole suspension	Drug interaction	Check of Posaconazole level, if below target level, stop pantoprazole if possible	Drug monitoring
Cotrimoxazole	23	No dose adjustment after weight gain	Subtherapeutic dosage	Dose increase based on body weight	Dose adjustment
Enalapril	18	Risk for hyperkalemia together with cyclosporine A and cotrimoxazole	Need for drug monitoring	Check of potassium level	Drug monitoring
Cyclosporine A	18	Application of Cyclosporine A together with CYP3A4 inhibitors/inducers	Drug interaction	Check of cyclosporine A level, dose adjustments may be necessary	Drug monitoring
Voriconazole	15	Phototoxicity	Side effect	Switch to Posaconazole	Drug switch
Posaconazole	13	Posaconazole inhibits the metabolism of CYP3A4-metabolized drugs	Drug interaction	Dose reduction of CYP3A4-metabolized drug based on plasma level or efficacy	Dose adjustment
Ondansetron	11	QTc prolongation together with other drugs potentially prolonging QTc interval	Side effect	If possible, stop or change drugs potentially prolonging QTc interval	Drug discontinuation
Amikacin	11	No dose adjustment to renal function in patient with renal impairment	Supratherapeutic dosage	Dose reduction based on renal function	Dose adjustment
Caspofungin	11	Dose is below recommended licensed dose	Subtherapeutic dosage	Regular dose in children is 50 mg/m^2^, max 70 mg/day	Dose adjustment
Furosemide	10	Hypochloremia together with etacrynic acid	Side effect	Additive side effect. Change drug or monitor, depending on severity of hypochloremia	Specific information

## Data Availability

All data and materials support the published claims and comply with field standards.

## Abbreviations

CP	Clinical pharmacist
CPS	Clinical pharmacy services
DRP	Drug-related problems
HSCT	Hematopoietic stem cell transplantation
PI	Pharmaceutical interventions
